# Factors associated with healthcare avoidance among transgender women in Argentina

**DOI:** 10.1186/s12939-014-0081-7

**Published:** 2014-09-27

**Authors:** María Eugenia Socías, Brandon DL Marshall, Inés Arístegui, Marcela Romero, Pedro Cahn, Thomas Kerr, Omar Sued

**Affiliations:** Fundación Huésped, Angel Peluffo 3932, Buenos Aires, C1202ABB Argentina; Department of Epidemiology, Brown University School of Public Health, 121 South Main Street, Box G-S-121-2, Providence, RI 02912 USA; Center for Psychology Research, School of Social Sciences, University of Palermo, Mario Bravo 1259, Buenos Aires, C1175ABW Argentina; Association of Transvestites, Transsexuals, and Transgenders of Argentina (A.T.T.T.A.), Callao 339 6th floor, Buenos Aires, C1022AAD Argentina; British Columbia Centre for Excellence in HIV/AIDS, St. Paul’s Hospital, 608-1081 Burrard Street, Vancouver, BC V6Z 1Y6 Canada; Department of Medicine, University of British Columbia, St. Paul’s Hospital, 608-1081 Burrard Street, Vancouver, BC V6Z 1Y6 Canada

**Keywords:** Transgender women, Argentina, Healthcare access, Police violence, Discrimination, Stigma

## Abstract

**Introduction:**

Transgender (TG) women in many settings continue to contend with barriers to healthcare, including experiences of stigma and discrimination. Argentina has a universal health care system and laws designed to promote healthcare access among TG women. However, little is known about barriers to healthcare access among TG women in this setting. The aim of this study was to explore individual, social-structural and environmental factors associated with healthcare avoidance among TG women in Argentina.

**Methods:**

Data were derived from a 2013 nation-wide, cross-sectional study involving TG women in Argentina. We assessed the prevalence and factors associated with avoiding healthcare using multivariable logistic regression.

**Results:**

Among 452 TG women included in the study, 184 (40.7%) reported that they avoided seeking healthcare because of their transgender identity. In multivariable analysis, factors positively associated with avoiding seeking healthcare were: having been exposed to police violence (adjusted odd ratio [aOR] = 2.20; 95% CI: 1.26 – 3.83), internalized stigma (aOR = 1.60, 95% CI: 1.02–2.51), having experienced discrimination by healthcare workers (aOR = 3.36: 95% CI: 1.25 – 5.70) or patients (aOR = 2.57; 95% CI: 1.58 – 4.17), and currently living in the Buenos Aires metropolitan area (aOR = 2.32; 95% CI: 1.44 – 3.76). In contrast, TG women with extended health insurance were less likely to report avoiding healthcare (aOR = 0.49; 95% CI: 0.26 – 0.93).

**Conclusions:**

A high proportion of TG women in our sample reported avoiding healthcare. Avoiding healthcare was associated with stigma and discrimination in healthcare settings, as well as police violence experiences. Although further research is warranted, these finding suggests that socio-structural interventions tailored TG women needs are needed to improve access to healthcare among this population.

## Introduction

Transgender (TG) women, the term for individuals who were assigned male sex at birth, but assume a feminine gender expression or identity, often experience multiple forms of oppression for transgressing gender norms, such as stigma, discrimination, isolation and economic hardship [1,2]. This marginalization, that is consequence of family, social and institutional transphobia, contributes to an increased risk of mental health problems, substance use and sexually transmitted infections. Collectively, these problems reinforce and perpetuate a harmful cycle of marginalization and poor health outcomes within this population [2-5].

Further, the widespread discrimination that TG women face, expose them to different forms of violence, which ranges from harassment, bullying, verbal abuse, physical violence to sexual assault and hate crimes [6-9]. In addition, due to the marginalization that many TG women experience, they interact frequently with police. Arbitrary arrest and detentions, which are common among this population, have been reported as an excuse to exploit TG women for bribes or coerce them into providing sex in exchange for release from detention [7,9-11].

TG women also face socio-structural inequalities within the health care system. Studies consistently show that TG individuals experience multiple challenges when attempting to access both routine and transition-related medical care, including denial of care, harassment, and lack of competent and sensitive providers with adequate knowledge of their specific needs [1,10,12-15]. As a result of these barriers, many TG women postpone or avoid urgent and medical care altogether. This, in turn, may help explain the poor health outcomes observed among this population, such as increased risk for HIV infection, substance use, and suicide attempts [3,10,15-18]. Indeed, despite the high burden of HIV infection among this population [19], studies show low rates of HIV testing [20], as well as poor outcomes at each step of the HIV continuum of care [21-23]. Furthermore, due to barriers to transition-related medical care, use of non-prescribed hormones or injection of industrial silicone in non-sterilized environments is widespread among TG communities, posing additional risks for their health [24,25].

Argentina has a universal but mixed health system that is composed by 3 sub-systems: (a) the public, (b) the social security which provides health coverage for formally employed workers and their families, and (c) the private sector. The Ministry of Health oversees all three subsectors. The network of public hospitals and health centers is open to anyone (including foreigners) and nominally free of charge. However, the public sector mostly provides care to low-income individuals without other forms of health insurance coverage, approximately 30% of the population [26,27]. Concerns persist regarding various social and structural factors that may constrain access to health services among TG individuals in this setting, thus significantly impacting their health. For example, life expectancy of TG women is approximately 35 years (compared to 79 years in other women) [28]. Furthermore, HIV prevalence among TG women in Argentina is estimated to be 34.1%, compared to 0.4% in the general population [29,30]. Other infectious diseases, such as syphilis, tuberculosis and viral hepatitis, are also more frequent among TG women [30,31], contributing to higher morbidity and mortality in this population. In response to this, and other related public health and social challenges, Argentina passed a progressive “Gender Identity Law” in May 2012 [32]. This law acknowledges the right to self-defined gender identity, allowing for changes to gender, image, or birth name on one’s identity card (ID), and ensures the right to appropriate transgender health services.

Despite these ongoing challenges and novel policy developments, little is known about the specific barriers to healthcare among TG women in Argentina, including those factors that may lead some TG women to avoid healthcare altogether. Therefore, the aim of this study was to explore potential individual, socio-structural and environmental factors associated with healthcare avoidance among TG women in Argentina.

## Methods

Data for the present analysis were derived from a nation-wide, semi-structured, cross-sectional survey, conducted by Fundación Huésped and A.T.T.T.A. (Association of Transvestites, Transsexuals, and Transgenders of Argentina), between June 2013 and December 2013. The objective of this survey was to collect baseline data and validate an instrument designed to assess the impact that the Gender Identity Law has had on living conditions among transgender individuals in Argentina.

In order to maximize representativeness of the transgender population in Argentina, snowball sampling was combined with quota sampling. As there are no official estimates of the size of the TG population in Argentina, the quotas (by region, by age group, and by educational level) were calculated using statistics provided by the national registration office (Registro Nacional de las Personas, RENAPER); specifically, the number of new IDs issued since the implementation of the “Gender Identity Law” and other national reports of socio-demographic characteristics of transgender individuals in Argentina. The goal was to recruit 450 TG women and 50 TG men.

Recruitment was done by peer outreach efforts facilitated by A.T.T.T.A. Recruitment venues included sex work areas and community-based organizations, among other places known to be frequented by TG individuals. Self-identified transgender individuals were eligible to participate. After providing written informed consent, enrolled individuals completed an interviewer-administered questionnaire. The surveys were conducted in private rooms by transgender peers previously trained in interviewer-administered assessment methods. The study was approved by the institutional ethics committee of Fundación Huésped. Participation was voluntary, and upon completing participants received a $100 ARS reimbursement (approximately $10 USD) for their time and effort. All data were de-identified to maximize confidentiality.

The interviewer-administered questionnaire captured information regarding socio-demographic characteristics, gender transition, HIV status, violence, interactions with police, healthcare access, housing, education, work, and experiences of stigma and discrimination in these settings.

For the current study, only TG women were included, and the primary outcome of interest was avoidance of healthcare due to transgender identity, defined as answering “Yes” to the following question: “Have you ever avoided going to a hospital or clinic because of your transgender identity?” With the selection of this dependent variable, we sought to measure and characterize avoidance of healthcare among TG women within Argentina’s universal healthcare system, which allows for analyses that are free from the confounding effects of affordability of health services.

In accordance with the Risk Environment Framework [33,34], we have sought to identify a range of individual, social-structural and environmental correlates of healthcare avoidance. Specifically, the following factors were explored:

- *Individual level factors:* age (less than versus greater than the median age), place of birth (Argentina versus other), having a job other than sex work (yes versus no), history of sex work involvement (yes vs. no), extended health insurance, defined as having either social security or private health coverage in addition to the universal public health coverage (yes versus no), high school education or higher (yes versus no), self-reported HIV infection status (yes, no, or unknown), and internalized stigma, defined as responding “yes” to any of the following questions: “have you ever felt any of the following emotions because of your transgender identity: ashamed, guilty, low self-esteem, feel that you should be punished”). Cronbach’s alpha for this stigma measure was 0.61.
- *Social-structural factors:* we asked participants to report if they had ever been arrested (yes versus no), and/or ever experienced violence from the police (defined as answering “yes” to any of the following questions: “Have you ever been exposed to any of the following situations due to your transgender identity: a policeman threatened you, a policeman beat, kicked or physically hurt you, a policeman forced you to have sex against your will?”). Cronbach’s alpha for this measure was 0.82. We also examined whether participants reported ever experiencing any perceived discrimination due to their transgender identity by either healthcare workers (including physicians, psychologists, social workers, and other administrative staff), or by patients (yes versus no). For the latter questions, and to provide contextualization, participants were provided with examples of specific situations of discrimination within the healthcare setting (e.g., denied health services, not called by their preferred name, mockery or threats).
- *Environmental factors:* residency (Buenos Aires metropolitan area, the biggest urban center in Argentina, vs. other), and current housing status (stable versus others).

Chi-square (for categorical variables) and Mann–Whitney (for continuous non-normally distributed variables) tests were conducted to compare TG women who reported avoiding or not healthcare. Bi- and multivariable logistic regression analyses were performed to examine potential associations between each independent variable and avoidance of healthcare. Variables found to be associated with the outcome at *p* < 0.10 in bivariable analyses were included in a multivariable logistic regression model. All associations were considered statistically significant at the two-tailed *p*-value < 0.05 threshold. A complete case analysis approach was employed, where cases with missing observations were excluded from the multivariable analyses. Analyses were performed using Stata/SE version 11.1 (Stata Corp, College Station, Texas).

## Results

Overall, 452 self-identified TG women completed the survey and were included in the study. Baseline characteristics of the participants are presented in Table 1. The majority of them were born in Argentina (90.0%), and had a median age of 30 years (interquartile range: 25–37). Three hundred and seventy eight participants (84.6%) reported a history of sex work (61.1% were currently engaged in sex work), and among those with previous HIV testing (n = 380, 84.1%), 27.4% reported being HIV-infected. As we have previously reported, characteristics among HIV-positive and HIV-negative TG women were similar [35]. The only significant difference was that compared to HIV-negative participants, TG women who self-reported HIV infection were more likely to be living in unstable housing conditions or had experienced police violence. Overall, 184 respondents (40.7%) reported that they had ever avoided seeking healthcare because of their transgender identity.

**Table 1:** **Baseline characteristics of transgender women included in the study, by whether they reported avoiding healthcare (N = 452)**

**Characteristic**	**Total (%)**	**Healthcare avoidance**		***p*** **- value**
	**(** ***n*** = 452)*	**Yes (** ***%*** **)**	**No (%)**	
		**(** ***n*** = 184)*	**(** ***n*** = 268)*	
*Individual level factors*				
**Age (median, IQR)**	30 (25 – 37)	30 (25 – 27)	31 (24 – 37)	0.861
**Foreign born**	45 (10.0)	17 (9.2)	28 (10.5)	0.673
**High school education or greater**	152 (33.8)	54 (29.5)	98 (36.7)	0.113
**Extended health insurance**	81 (18.5)	25 (13.8)	56 (21.7)	0.036
**Currently employed (other than sex work)**	108 (23.9)	35 (19.0)	73 (27.2)	0.044
**History of sex work involvement**	378 (84.6)	163 (89.1)	215 (81.4)	0.028
**Self-reported HIV infection**	104 (27.4)	41 (26.8)	63 (27.8)	0.838
**Any internalized stigma**	245 (54.2)	118 (64.1)	127 (47.4)	<0.001
*Social-structural factors*				
*Police-related experiences*				
**Experienced police violence ever**	243 (53.8)	128 (69.6)	115 (42.9)	<0.001
**Ever arrested**	354 (79.0)	157 (85.8)	197 (74.3)	0.003
*Experiences of perceived discrimination in healthcare settings*				
**By healthcare workers ever**	302 (66.8)	155 (84.2)	147 (54.9)	<0.001
**By other patients ever**	143 (32.1)	84 (46.4)	59 (22.4)	<0.001
*Environmental factors*				
**Current residency in Buenos Aires**	140 (31.0)	69 (37.5)	71 (26.5)	0.013
**Stable housing**	355 (78.6)	143 (77.7)	212 (79.1)	0.724

*Totals may differ due to non-response on some questions.

IQR: interquartile range.

Table 2 shows the results of the unadjusted and adjusted analyses of potential correlates of healthcare avoidance among TG women in our sample. Factors significantly and positively associated with avoidance of healthcare in bivariable analysis included: currently living in the Buenos Aires metropolitan area, having a history of sex work, experiencing internalized stigma as a result of one’s gender identity, having ever been arrested, having been exposed to police violence (e.g., threats, beating, or sexual abuse), and having experienced discrimination in healthcare settings, both by healthcare workers and by patients (all *p* < 0.05). In contrast, TG women with extended health insurance or a job other than sex work were less likely to report avoidance of healthcare (both *p* < 0.05).

**Table 2:** **Bi- and multivariable logistic regression analyses of individual, environmental and socio-structural factors associated with healthcare avoidance among transgender women in Argentina**

**Characteristic**	**Odds ratio (OR)**	
	**Unadjusted OR (95% ** **CI)**	**Adjusted OR (95% ** **CI)**
*Individual level factors*		
**Age ≥ 30 years old**	1.02 (0.70 – 1.48)	—
**Foreign born**	0.87 (0.46 – 1.64)	—
**High school education or greater**	0.72 (0.48 – 1.08)	—
**Extended health insurance**	0.58 (0.35 – 0.97)*	0.49 (0.26 – 0.93)
**Currently employed (other than sex work)**	0.63 (0.40 – 0.99)*	1.00 (0.56 – 1.79)
**History of sex work involvement**	1.86 (1.06 – 3.25)*	0.86 (0.41 – 1.80)
**Self-reported HIV status**	0.95 (0.60 – 1.51)	—
**Any internalized stigma**	1.98 (1.35 – 2.92)*	1.60 (1.02 – 2.51)
*Social-structural factors*		
*Police-related experiences*		
**Experienced police violence ever**	3.04 (2.05 – 4.52)*	2.20 (1.26 – 3.83)
**Ever arrested**	2.08 (1.27 – 3.43)*	1.00 (0.48 – 2.11)
*Experiences of perceived discrimination in healthcare settings*		
**By healthcare workers ever**	4.40 (2.77 – 7.00)*	3.36 (1.25 – 5.70)
**By other patients ever**	3.01 (1.99 – 4.54)*	2.57 (1.58 – 4.17)
*Environmental factors*		
**Current residency in Buenos Aires**	1.66 (1.11 – 2.49)*	2.32 (1.44 – 3.76)
**Stable housing**	0.92 (0.58 – 1.45)	—

*Significant at *p <* 0.10 and entered into the multivariable model.

CI: confidence interval.

As indicated in Table 2, factors that remained independently and positively associated with avoiding seeking healthcare in the multivariable analysis were having been exposed to police violence (adjusted odd ratio [aOR] = 2.20; 95% CI: 1.26 – 3.83), internalized stigma (aOR = 1.60, 95% CI: 1.02–2.51), having experienced discrimination by healthcare workers (aOR = 3.36: 95% CI: 1.25 – 5.70) or patients (aOR = 2.57; 95% CI: 1.58 – 4.17), and currently living in the Buenos Aires metropolitan area (aOR = 2.32; 95% CI: 1.44 – 3.76). Having extended health insurance remained significantly and negatively associated with the outcome (aOR = 0.49; 95% CI: 0.26 – 0.93).

## Discussion

In this cross-sectional study, we found that approximately 40% of TG women reported avoiding healthcare because of their transgender identity. Reports of avoiding healthcare were found to be independently associated with internalized stigma, having experienced discrimination by healthcare workers or patients, having been exposed to police violence, and currently living in the Buenos Aires metropolitan area. In contrast, TG women with extended health insurance were less likely to report avoiding healthcare. These findings raise concern about the role of various social-structural factors in shaping access to healthcare among TG women, as well as concerns about suboptimal access to care among a population that is contending with various risks, including ongoing high rates of HIV infection [19].

Previous studies have documented constrained access to healthcare among transgender communities throughout the globe [8,13,14,17,36-39]. Challenges that transgender women face when trying to access healthcare include inappropriate or nonexistent care protocols for transgender clients, as well as untrained and often discriminatory attitudes among health providers and staff [10,12,14,40]. These challenges, in turn, may contribute to TG women’s perceptions of poor quality of and disrespectful care, which further shape their healthcare seeking behaviors. Our findings are consistent with previous studies, as we found that experiencing internalized stigma and having suffered discrimination in healthcare settings were positively associated with avoiding healthcare in multivariable analysis. Choosing not to seek medical care could be a way in which TG women cope with low levels of availability of gender-competent care and avoid further experiences of discrimination. Accordingly, training healthcare providers to ensure greater knowledge of and sensitivity to transgender health issues is critical. Wherever possible, transgender individuals should be involved in these initiatives.

Interestingly, the results of this study show that discrimination within healthcare facilities may come not only from healthcare providers and staff, but also from patients, which could be a reflection of the pervasiveness of transphobia within the larger society. Supporting this finding, a nation-wide qualitative study conducted by the Ministry of Health among 218 LGTB individuals (57 transgender individuals) showed that experiences of stigma and discrimination among this group are widespread, not only within the healthcare setting, but also in educational, work, and other public settings [7]. Altogether, these findings suggest that other interventions, besides educating healthcare providers, might be needed in order to achieve culturally competent transgender friendly health care services. Increasing employment opportunities for transgender individuals in healthcare settings could have the dual benefit of creating more job opportunities for this population, as well as helping to create a more welcoming environment for transgender individuals seeking medical care [3].

We also found a strong and positive association between experiences of police violence and avoidance of healthcare. Specifically, TG women may choose not to seek healthcare as a way to avoid further negative interactions with police or other personnel (e.g., security guards) in these environments. This finding is consistent with a large and growing body of literature pointing to the important role that policing practices can play in shaping the health of marginalized populations at risk for or living with HIV disease. A previous study of female sex workers in Vancouver, Canada, showed that policing presence and violence often displaces sex workers into more remote locations that are far away from health programs [41]. Other studies have shown that fear of police can foster reluctance among people who use drugs to access HIV prevention programs or carry sterile syringes [42-44]. To our knowledge, this is the first study to reveal a relationship between police violence and the avoidance of healthcare among TG women. This finding is particularly concerning given the frequent confrontations with police among TG women, as demonstrated by the high rates of TG women in our sample who have been arrested (79%) or experienced different forms of police violence (54%). Future research should seek to elucidate the individual-level interactions and structural mechanisms that may explain this association, to better understand how police violence mediates healthcare access in this population. Regardless, efforts should be made to: ensure more effective monitoring of and sanctions for police misconduct; encourage systems for reporting and redress among victims; and provide educational approaches for police that focus on the unique experiences and needs of TG women.

Similar to previous research in other settings [36-38], we also found that TG women with extended health insurance were less likely to avoid seeking healthcare. This finding suggests that TG women lacking extended health insurance (as a proxy for low socio-economic status) may have other competing interests (e.g., money, food sources, distance, time) that could make seeking healthcare a low priority, even within a universal health care system. Therefore, additional efforts are needed to reach TG women with lower socio-economic status, and ensure access to appropriate and high-quality healthcare services in all 3 sub-sectors.

Finally, TG women living in the Buenos Aires metropolitan area (the biggest urban center in Argentina), were more likely to report avoiding healthcare. While we cannot exclude unmeasured confounding factors, such as frequent concentration of persons of lower socioeconomic status, as well as large migrant and minority populations in cities, this association persisted in the multivariable analysis. A possible explanation is that TG women residing in smaller cities may be more likely to have family support and tighter bonds with their communities, which can facilitate their access to and navigation through the health system. Alternatively, it might also be possible that TG women in Buenos Aires are more likely to be exposed to or engaged in the large transgender sex work scene in this city, and are therefore also more likely to experience police violence than those TG women living in smaller cities or towns. Indeed, previous research has described the relationship between sex work and drug scenes and police presence and misconduct [43,45]. Further work is needed to examine specific reasons for healthcare avoidance behaviors among TG women in Buenos Aires, specifically.

Collectively, these findings add to the growing body of evidence highlighting the importance of social, structural and environmental factors as drivers of disease burden and healthcare access among marginalized populations, such as injection drug users, sex workers, and transgender individuals [2,16,34,46,47]. Policy-level interventions that protect transgender rights have potential to improve access to health care among this population. The recently passed Argentinean “Gender Identity Law” [32] establishes a framework based on equity and human rights, and acknowledges the right to self-defined gender identity. Unlike many settings [48], this law allows for changes to gender, image, or birth name on one’s identity card and birth certificate without a requirement of psychiatric evaluation or judge approval. Since the enactment of this law in May 2012, over 3,000 TG individuals have acquired new government issued identification [49]. This law further supports the right to the full development of one’s person in line with one’s chosen gender identity, and ensures the right to appropriate health services. For many TG women, body modification procedures are an important part of one’s gender identity affirmation, and therefore increasing access to transition-related medical care could potentially help to engage and retain TG women in care. However, the impact of this new law remains under-evaluated, and further research is required to examine its health and social benefits in TG populations in Argentina.

Our study has a number of limitations. First, as there are no official registries of TG women in Argentina, our sample was not randomly selected, and therefore we cannot assume that our results are generalizable to all TG women in Argentina. We tried to mitigate this potential source of bias by recruiting a large sample, and by using a sampling quota technique to ensure the recruitment of TG participants from different age groups, educational levels and regions. Nevertheless, the possibility of non-random sampling bias remains. Second, our analysis relied on self-reported data, which may be susceptible to recall and social desirability biases. Third, although we followed and adapted previous validated instruments to measure internalized stigma among marginalized populations in Argentina such as the HIV Stigma and discrimination Index [50], and despite the questionnaire design was informed by previous focus groups [51] and tested by the transgender interviewers themselves, the internal consistency of our constructed “internalized stigma variable” was only acceptable (α = 0.61). Fourth, another limitation of our study is that participants may hold diverse understandings of the term “healthcare”, and because we did not assess specifically what type of medical care (e.g., curative or preventive, minor or major illnesses) the participants avoided, we were unable to further contextualize this health behavior. Fifth, as the study is cross-sectional, and we included both lifetime and current explanatory variables (and the outcome referred to a lifetime behavior), temporality and causal associations could not be determined. Sixth, responses to many of the key variables of interest were limited to simple forced-choice responses (e.g., yes/no), which have may have precluded more detailed and in-depth responses. Accordingly, the findings presented here should be explored in greater detail through in-depth qualitative and ethnographic research. Lastly, although we performed multivariable analysis to adjust for known relevant confounders, there may be unmeasured factors (e.g., substance abuse) that may confound the relationship between exposures and outcomes.

## Conclusions

In summary, we found that 40% of TG women in our sample reported avoiding healthcare due to their transgender identity. Despite the limitations acknowledged, our study provides important information regarding contextual factors shaping healthcare access among TG women in Argentina. Of particular concern is the finding that, aside from the stigma and discrimination experienced within the healthcare setting, police violence was also independently associated with healthcare avoidance. Given the high burden of disease, and the high rates of discrimination and violence experienced by TG women in Argentina, there is an urgent need to adapt and develop socio-structural interventions that are tailored to promote healthcare access among this vulnerable population.
